# Pharmacokinetics and pharmacodynamics of the cytolytic anti‐CD38 human monoclonal antibody TAK‐079 in monkey – model assisted preparation for the first in human trial

**DOI:** 10.1002/prp2.402

**Published:** 2018-05-17

**Authors:** Stefan Roepcke, Nele Plock, Josh Yuan, Eric R. Fedyk, Gezim Lahu, Lin Zhao, Glennda Smithson

**Affiliations:** ^1^ Takeda Pharmaceuticals International AG Zurich Switzerland; ^2^ Takeda Pharmaceuticals International Cambridge Massachusetts

**Keywords:** anti‐CD38 antibody, cytolytic antibody, lymphocyte depletion, PKPD modeling

## Abstract

We are studying the fully human, IgG1λ cytolytic monoclonal antibody TAK‐079, which binds CD38. CD38 is expressed on plasma and natural killer (NK) cells constitutively and upregulated on subsets of B and T lymphocytes upon activation. TAK‐079 cross‐reacts with CD38 expressed by cynomolgus monkeys and depletes subsets of NK, B, and T cells. Therefore, safety and function of TAK‐079 was evaluated in this species, prior to clinical development, using bioanalytical, and flow cytometry assays. We pooled the data from eight studies in healthy monkeys (dose range 0.03‐100 mg/kg) and developed mathematical models that describe the pharmacokinetics and the exposure–effect relationship for NK cells, B cells, and T cells. NK cell depletion was identified as the most sensitive pharmacodynamic effect of TAK‐079. It was adequately described with a turnover model (*C*_50_ *= *27.5 μg/mL on depletion rate) and complete depletion was achieved with an IV dose of 0.3 mg/kg. Intermediate effects on T‐cell counts were described with a direct response model (*C*_50_ *= *11.9 μg/mL) and on B‐cell counts with a 4‐transit‐compartment model (*C*_50_ *= *19.8 μg/mL on depletion rate). Our analyses substantiate the observation that NK, B and T cells are cleared by TAK‐079 at different rates and required different time spans to replete the blood compartment. The models were used to simulate pharmacokinetic and cell depletion profiles in humans after applying a straightforward scaling approach for monoclonal antibodies in preparation for the first‐in‐human clinical trial.

**Abbreviations**
ADA: anti‐drug antibody
AUC: area under the concentration‐time curve
BASE: baseline B‐cell levels
BSV: between subject variability
CRP: C‐reactive protein
ECL: electrochemiluminescence
ELISA: enzyme‐linked immunosorbent assay
FIH: first‐in‐human
GLP: good laboratory practice
GOF: goodness of fit
IQR: interquartile range
LLOQ: lower limit of quantification
MOEF: molecules of equivalent soluble fluorescence
NCA: noncompartmental analysis
NCA: noncompartmental analysis
OFV: objective function value
PD: pharmacodynamics
PK: pharmacokinetics
QC: quality control
QSS: quasi steady state
RT: room temperature
TCM: transit compartment model
TMDD: target‐mediated drug disposition

## 1 INTRODUCTION

TAK‐079 is a fully human IgG1λ monoclonal antibody with high affinity for CD38 that is developed for the treatment of multiple myeloma and autoimmune diseases.^2^, ^5^, ^14^, ^21^, ^22^ The target molecule CD38 is a type II‐transmembrane protein which functions as a cell adhesion receptor and a multifunctional ectoenzyme.^8^, ^18^, ^20^ It is highly expressed on plasmablasts, plasma cells, NK cells, and activated T and B cells in normal healthy subjects and on malignant plasma cells in multiple myeloma patients.^18^ In vitro models with human cell lines demonstrated that binding of TAK‐079 to CD38 induced depletion of human B cell lines by antibody‐dependent cell‐mediated cytotoxicity and complement‐dependent cytotoxicity and, in most cases, cell lines with increased CD38 expression were more susceptible to cell lysis.^22^

The amino acid sequences of human and rodent CD38 exhibit low homology, whereas the homology of the human CD38 protein with cynomolgus monkeys is considerably higher (92%).^7^, ^13^, ^16^ Despite the high homology, the anti‐human CD38 monoclonal antibodies daratumumab (Darzalex) and isatuximab do not cross‐react with monkey CD38.^4^, ^26^ In contrast, TAK‐079 binds to monkey CD38 and this provides the unique opportunity to study anti‐CD38 cytolytic activity in nonhuman primates.

The objectives of this study were to characterize the pharmacokinetics (PK) and pharmacodynamics (PD) of TAK‐079 in monkeys and to build mathematical models, which could guide dose selection for the first‐in–human (FIH) clinical trial. To this end, assays were developed to measure drug concentrations and immunogenicity, and to quantify T, B, and NK lymphocytes in the blood of monkeys. We assessed these parameters in eight pharmacological and toxicological preclinical studies. Mathematical models that describe the PK and PD data of therapeutical monoclonal antibodies were recognized as useful tools to gain mechanistic and quantitative insights into the relationships between drug exposure and effect.^9^, ^12^, ^17^ Typical PK features of IgG antibodies including distribution and elimination, physiological and genetic similarities between monkey and human could be leveraged to explain the pharmacology of TAK‐079.^11^, ^15^ In addition, those models have been successfully applied to predict PK concentrations and PD effects in healthy human subjects.^12^ Here we describe the derivation of unique PK and PD models of anti‐CD38 activity and the first opportunity to utilize this model for guiding the design of the first in human studies of anti‐CD38 therapeutics.

## 2 MATERIALS AND METHODS

### 2.1 Animal studies

The studies were conducted in cynomolgus monkeys (Macaca fascicularis). A summary of them is shown in Table 1 in chronological order. The single dose studies 2, 7, and 8 were primarily conducted to evaluate PK and PD of intravenously (IV) and subcutaneously (SC) administered TAK‐079. The repeated dose studies were performed to evaluate safety, PK, and PD, including two 4‐week studies (studies 1 and 3) and three 13‐week studies under Good Laboratory Practice (GLP) conditions (studies 4, 5, and 6). In study 4 seven doses of 3, 30 or 80 mg/kg were administered every other week (Q2W). According to the protocol the majority of animals were terminated after 98 days (14 days after the last dose) for detailed toxicological assessments. 4 animals of each group were assigned to a recovery group and kept for additional 98 days. In the other 13‐week studies 5 and 6, the animals received weekly doses (QW). In study 5, in which a dosing error occurred, animals in the lowest dose group received 0.01 mg/kg instead of the intended 0.1 mg/kg at one occasion (the second dose) and then continued with 0.1 mg/kg. These data were added to the data set with the correct information of the actual administered dosing amounts. Study 6 repeated the low dose of 0.1 mg/kg QW group of study 5. All animal studies were carried out in accordance with the Guide for the Care and Use of Laboratory Animals as adopted and promulgated by the U.S. National Institutes of Health.

**Table 1:** TAK‐079 monkey studies in chronological order

Study	Study description	Number of animals (female, male)	Dose levels (mg/kg)	Number of samples per animal (PK/PD)^a^
1	Day 1 (1 mg/kg) + Day 28 (2 mg/kg), IV, PK, PD	6 (0, 6)	plc, 1, 2	19/10
2	Single dose, IV, PK, PD, ADA	9 (0, 9)	plc, 0.3, 3	14/9
3	4 weeks tox, QW, IV, PK, PD, ADA	12 (4, 8)	plc, 1, 30, 100	15/8
4	13 weeks tox, Q2W, IV, PK, PD, ADA	40 (20, 20)	plc, 3, 30, 80	28/17
5	13 weeks tox, QW, IV, PK, PD, ADA	52 (26, 26)	plc, 0.1, 0.3, 1	31/9
6	13 weeks tox, QW, IV, PK, PD, ADA	20 (20, 0)	plc, 0.1	31/10
7	Single dose, IV/SC, PK, PD, ADA	12 (12, 0)	0.1, 0.3, 1	16/16
8	Single dose, IV/SC, PK, PD	24 (24, 0)	0.03, 0.1, 0.3	19/19

IV, intravenous 30 minutes infusion (studies 1‐4) or bolus (studies 5‐8); SC, subcutaneous injection (single group of study 7 and 3 groups in study 8); PK, blood sampling for the assessment of TAK‐079 serum concentrations; PD, whole blood sampling for flow cytometry analyses yielding cell count data of T, B, and NK cells; ADA, anti‐TAK‐079 antibody assessment; plc, placebo; “4 weeks” or “13 weeks” describe the duration of the treatment period; tox, toxicology study; QW, weekly dosage; Q2W, every other week dosage.

a Sample numbers are approximate or maximum numbers according to the protocol.

### 2.2 Bioanalytics

PK was analyzed using a validated method developed and performed by Charles River Laboratories (Reno, NV). Briefly, the concentration of TAK‐079 was measured in monkey serum, using an indirect enzyme‐linked immunosorbent assay (ELISA). A 96‐well plate was coated with anti‐idiotypic antibody against TAK‐079. Blanks, standards, and quality control (QC) samples containing TAK‐079 at various concentrations were added to the plate, and incubated for 5565 minutes at room temperature (RT). After washing the plate, the detection antibody (Peroxidase AffiniPure Mouse Anti‐Human IgG, Fcγ Fragment Specific; Jackson ImmunoResearch) was added, and incubated on the plate for an additional 5565 minutes. The plate was washed, and tetramethylbenzidine was added to the wells to generate a chromophore, and the reaction stopped by the addition 2N sulfuric acid. Absorbance at 450 nm was measured using a SPECTRAmax^®^ 190 microplate reader (Molecular Devices) and the TAK‐079 concentrations calculated using a 4‐parameter logistic weighted (1/y^2^) standard calibration curve. In the first study (Table 1), the lower limit of quantification (LLOQ) of TAK079 in serum was 0.061 μg/mL and in all other studies it was 0.05 μg/mL.

### 2.3 Determination of anti‐TAK‐079 antibodies (immunogenicity)

Anti‐drug antibodies (ADA) screening of monkey serum was done, using qualitative electrochemiluminescent (ECL) method, validated and performed by Charles River Laboratories (Reno, NV). Briefly, undiluted serum samples were mixed acid dissociated (300 mmol/L acetic acid) then incubated in a mixture of biotinylated TAK‐079, TAK‐079 labeled with SULFO‐TAG (Meso Scale Diagnostics, labeled at Charles River) and 1.5 mol/L Trizma base to neutralize the acid and form an immune complex. This complex was added to a streptavidin‐coated MSD plate (Meso Scale Diagnostics) and allowed to bind. After washing, the complex was detected by the addition of MSD read buffer T (Meso Scale Diagnostics) to the plate and subsequent excitation was read, using the MSD Sector 6000 (Meso Scale Diagnostics).

### 2.4 Characterization of blood cells

To compare the level of TAK‐079 binding between humans and monkeys, blood samples from each were collected into sodium heparin tubes and an aliquot (100 μL) was mixed with appropriate volume of each antibody (Table S1) and incubated for 15‐20 minutes at RT. After incubation, 1 mL of BD FACS lyse (1X; BD Biosciences; San Jose, CA) was added to lyse red blood cells and the cells incubated for 10 minutes, centrifuged, and washed with staining buffer containing bovine serum albumin (BD Biosciences). The cells were resuspended in 250 μL of Flow Fix (1% paraformaldehyde in calcium and magnesium‐free Dulbecco's‐PBS (Life Technologies, Carlsbad, CA) and fluorescence measured with FACSCanto™ II Flow Cytometer (BD Biosciences). The median fluorescence intensity for a TAK‐079 staining for each cell population was converted into units of molecules of equivalent soluble fluorescence (MOEF), using a standard curve generated with Rainbow Beads (Spherotech; Lake Forest, IL).

In studies outlined in Table 1, cells were stained and analyzed, using a validated method developed and performed by Charles River Laboratories (Reno, NV). Monkey blood samples were collected into sodium heparin tubes before and at multiple times after TAK‐079 treatment and specific lymphocyte populations measured, using FACSCanto™ II Flow Cytometer (BD Biosciences). Commercial antibodies and a CD38 antibody (TSF‐19) were titered to optimal concentrations for staining. T‐cell, B‐cell and NK‐cell populations were identified and lymphocytes quantified, using CD45TruCount™ tubes (BD Biosciences). Blood cells (100 μL aliquots) were mixed with antibodies added at the indicated volume (Table S1), and incubated for a minimum of 30 minutes at RT. Red blood cells were lysed, samples mixed and incubated at RT for an additional 10 minutes. Cells were washed, and then resuspended in 500 μL of stain buffer with fetal bovine serum. In studies 1‐4 CD38+ NK, B, and T‐cell subsets were assessed at baseline with the labeled anti‐CD38 antibodies TAK‐079 or TSF‐19 (Table S1). Although TSF‐19 binds to a different epitope, the results were very similar and are therefore not presented separately.

### 2.5 PK model development

During PK model development, 1‐, 2‐, and 3‐compartment models were investigated. Note that the effect of ADA was not described in the model but the affected data points were excluded during modeling and parameter estimation. The two‐compartment model was clearly superior to the one‐compartment model, as judged by goodness‐of‐fit (GOF) plots and decrease in objective function value (OFV). Based on visual inspections of diagnostic plots, the introduction of a third compartment was not necessary to describe the data adequately. The bioavailability (*F*) was modeled using the logit transformation *F* = exp (PAR)/(1 + exp (PAR)), where PAR designates the model parameter, to ensure that the estimates are bounded between 0 and 1. The nonlinear PK at low concentrations was modeled with the QSS approximation model of the target‐mediated drug disposition (TMDD) process.^10^ A schematic representation and the differential equations of the final PK base model are provided in Figure 2. For the QSS approximation, we assume that the steady‐state concentrations of the free drug C, the target R, and the drug target complex RC are established very quickly compared to all other processes. This implies that the binding process is balanced with the dissociation and internalization processes and that the following equation holds in the appropriate units: *K*_ON_ * C * R = (*K*_OFF_ + *K*_INT_)*RC, where *K*_ON_ designates the binding rate constant and *K*_OFF_ the dissociation rate constant, and *K*_INT_ the internalization rate constant.

The between–subject variability (BSV) was investigated for all parameters and modeled with exponential models of the following type: PAR_*i*_ *= *TVPAR * e ^ETAPAR^_*i*_, where PAR_*i*_ is the individual and TVPAR the estimate of the typical value (or point estimate) of the parameter and ETAPAR_*i*_ is the estimate of the deviation of individual *i*. The ETAPAR_*i*_ values were assumed to follow a normal distribution with mean zero. The residuals were described with a combined additive and proportional error model.^1^

The following characteristics that could be potential covariates of the PK of TAK‐079 were available in the data set: body weight, sex, dose, route of administration, and study. Note that the actual dose of each animal was calculated based on the dose level (in mg/kg) and its predose body weight. The covariates were investigated by correlating their individual levels with the individual deviations of each of the PK parameters. Most of the correlations were negligible so that it was unlikely that the covariate level could explain significant parts of the between subject variability of the PK parameter. Only the potential effects of the route of administration were tested systematically in a stepwise inclusion procedure.

### 2.6 PK‐PD model development

For each of the three cell types, PK‐PD model development was performed separately, during which the PK model and parameter estimates were kept fixed. Note that for model development measurements close to the drug administration (<8 hours postdose) were not utilized because they were influenced by a nonspecific drug‐independent effect. Turnover, transit compartment and direct response models of various forms were tested.^9^, ^17^ In the turnover models, the drug effect was introduced on the cell elimination rate in form of an *E*_max_ type model with or without Hill factors. In our notation, an *E*_max_ model is a function *f* of the drug concentration *c* of the following form: *f(c)*_**_ *= E*_MAX_ * c^H^/(c^H ^+ *C*_50_^H^), *E*_MAX_ denotes the maximal effect, *C*_50_ the concentration at which half of the maximal effect is achieved, and H the Hill factor. In the transit compartment model (TCM), the drug effect was introduced and tested on distinct positions: on the rate of proliferation, on circulating cells and on the third transit compartment. Also, combinations of these effects and whether the data supports the presence of a feedback mechanism from the circulation to the rate of proliferation were also tested. In addition, Emax type direct response models with and without Hill factors were tested to describe the drug concentration effect curve.

Random‐effect parameters were introduced to estimate the between‐subject variability on the baseline cell count, on the cell production rate (*K*_IN_), on transit time in the transit compartment model (MTT), on *C*_50_ and on *E*_MAX_. Individual mean baseline cell levels were provided in the data set (column BL). This was used as typical value (individual point estimate) in the model. A random effect parameter was added to enable the adjustment of the individual baseline estimate based on all measurements of that animal. The PD residuals were described with a proportional error model.

For model validation during the modeling (PK and PK‐PD) OFV, standard errors, GOF plots and individual prediction versus data plots were used to assess the models and compare them to alternative ones.

The following software packages were utilized NONMEM (Version 7.3), KIWI (Version 1.6), Berkeley Madonna (version 8.3.14), PSN (Version 4), and R (Version 3.3.0). For the estimations in NONMEM, the subroutines ADVAN6 TOL_**_ *= *3 for the PK model and ADVAN13 TOL_**_ *= *5 for the PK‐PD models and the method first‐order conditional estimation with interaction FOCE (METHOD_**_ *= *1 INT) were used.

## 3 RESULTS

### 3.1 Pharmacokinetics of TAK‐079

**Figure 1:**
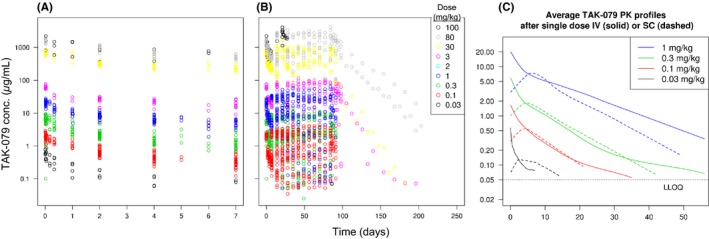
Monkey PK data of TAK‐079. Pannel (A) and (B) show the raw PK data of the 8 monkey studies, panel (A) the first 7 days after the first dose and panel (B) the entire observation period. Panel C shows the smoothed averaged PK profiles of the low first doses IV (solid curves) and SC (dashed curves) administration groups (averaging with R function *loess.smooth*)

The PK data set was pooled from all eight studies in healthy monkeys excluding the placebo groups (Table 1). In total, the set contained data from 140 animals (58 males and 82 females). The body weights of the studied animals ranged from 2.1 to 4.7 kg and the doses ranged from 0.03 to 100 mg per kg body weight (mg/kg). In one group of study 7 and three groups of study 8 doses of 0.03, 0.1, 0.3 and 1 mg/kg were administered subcutaneously (15 animals in total). The pooled data set contained 2,199 measurable PK observations greater than LLOQ (Figure 1A and B). In parallel to TAK‐079 PK concentrations the effects of anti‐drug immunogenicity on the PK was assessed by measuring ADA titers in 6 of the 8 studies. The ADA assays were not the same between the studies so that the resulting titers could not be directly compared quantitatively. However, exploratory analyses in the 13‐week toxicology study (study 5) confirmed the relationship between ADA titer and TAK‐079 concentration (Figure S3). Moreover, in the repeated dose studies it was obvious that the anti‐drug immunogenicity developed over time since the number affected animals and the individual titers increased. Therefore, we digitized the ADA information and added a present/absent flag to each observation (see Data Set Preparation in Supporting Information for details). In total, 229 PK observations were flagged to be affected by ADA so that it could be excluded during model development.

Initially, for each of the monkey studies PK analyses were performed using standard noncompartmental techniques (NCA). Based on the single dose studies (IV bolus injection or 30 min IV infusion) the volume of distribution during the terminal phase (*V*z) was calculated to range from 64 to 116 mL/kg, the clearance from 6.04 to 14.7 mL/kg per day, and the terminal elimination half‐life (T1/2) from 4.75 to 11.2 days. Area under the concentration time curve (AUC) and maximal concentration (*C*_max_) values were found to increase proportionally with dose over a wide range. Only the PK profiles of the lowest dose groups (<1 mg/kg, Figure S2) provide evidence for nonlinearly augmented clearance at concentrations below 0.5 μg/mL likely caused by target‐mediated mechanisms (TMDD).^15^

The PK data after single dose SC administration were generated in preparation for the first in human clinical trial using another formulation of TAK‐079 (Figure 1C). The data revealed that *C*_max_ was 70%‐80% lower in the SC versus IV groups of the same dose, and that there were no systematic differences in elimination or AUC between the groups.

No systematic differences in PK parameters between male and female monkeys were observed. The results of these initial analyses were used as the starting point for model development.

### 3.2 PK model development

**Figure 2:**
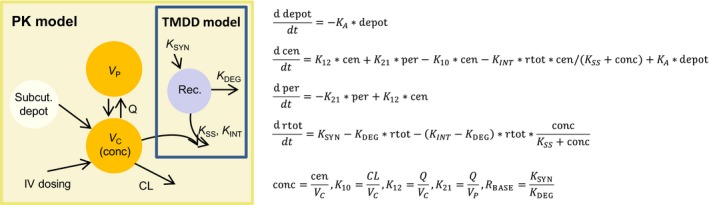
PK model structure and formulas. The final base PK model structure and formulas including TMDD marked with the blue box. *V*_C_ designates the volume of the central compartment where the TAK‐079 concentrations were observed (conc). V_P_ designates the volume of the peripheral compartment and Rec the compartment of the antibody bound and unbound receptor CD38. Parameter descriptions are given in Table 2. The differential equations specify the PK model in terms of amount of drug (*cen* and *per*) and total amount of receptor (*rtot*)

Model development started with single IV dose data and then the initial model was gradually extended utilizing more complex data. Similar to other therapeutic antibodies, the PK grossly follows a linear 2‐compartment model.^15^ The nonlinear elimination component (TMDD) describing the accelerated clearance at low concentrations was modeled with the QSS approximation.^10^ The assumption that the drug–target association process is much faster than the processes of drug dissociation, distribution and elimination, and of target and drug–target complex elimination leads to the simplified TMDD model (Figure 2, Table 2). The amount of data at low concentrations was relatively small and we did not manage to estimate all the parameters successfully in a single estimation run of the software program. Therefore, we chose to define the model structure and estimate the parameters of the TMDD model based on the data of the low single dose studies 7 and 8. The resulting TMDD model structure and parameter estimates (except the BSV of *K*_INT_) were then kept fixed during the final estimations on the entire data set (Table 2).

**Table 2:** Population PK modeling results: parameter estimates and standard errors in percent (%SE)

Parameter	Description	Final parameter estimate		Between subject variability/residual variability	
		Typical value (point estimate)	% SE	Magnitude	% SE
F	Bioavailability	0.227	121	NE	–
*K*_A_ (L/day)	Rate of absorption	0.399	20.5	42.1% CV	53.8
CL (L/day)	Clearance	0.0187	5.17	42.9% CV	20.1
*V*_C_ (L)	Central volume of distribution	0.141	3.23	19.8% CV	22.5
Q (L/day)	Intercompartimental clearance	0.127	14.7	NE	–
*V*_P_ (L)	Peripheral volume of distribution	0.127	6.45	39.4% CV	20.8
*K*_INT_ (1/day)	Complex elimination rate	0.1	FIXED	49.3% CV	36.7
*K*_SS_ (1/day)^a^	Steady‐state constant	5.68	38.7^c^	NE	–
*K*_SYN_ (u/L per day)^b^	CD38 synthesis rate	0.04	FIXED	NE	–
*K*_DEG_(1/day)	CD38 degradation rate	0.00452	30.1^c^	NE	–
ROUT on *V*_C_	Covariate effect	−0.697	6.51	NE	–
RV add	Residual variability (additive component)	–		3.17E‐04	21.2
RV prop	Residual variability (proportional component)	–	–	0.0677	1.58
	Minimum value of the objective function_* *_ *= *5748.412				

a *K*_SS_ is the steady‐state constant, defined as *K*_SS**_ *= (K*_OFF_ *+ K*_INT_ *)/K*_ON_, where *K*_OFF_ is the dissociation and *K*_ON_ the binding rate constant.

b *K*_SYN_ is the synthesis rate of the receptor CD38. Since we do not have actual concentration measurements or information about the in vivo synthesis or degradation rate of CD38 we use “u” as unit for a certain unknown amount of CD38. CV, coefficient of variation; RV, residual variability; NE, Not Estimated.

c The estimates and standard errors for the TMDD parameters were gained from a separate run that focused on the data of the low dose groups (residual variability of the separate estimation: additive 0.005, proportional 0.067), and were then fixed for the final estimation of the other PK parameters.

We obtained estimates for the absorption rate (*K*_A_) and the bioavailability (*F*) when we added the data of the SC groups. Note that all SC data come from four lower single dose groups from studies 7 and 8. These lower doses (≤1 mg/kg) cover the estimated clinically relevant range but may limit the generalizability of the parameter estimates for higher doses.

During the covariate analysis, we searched for potential relationships between body weight, sex and route of administration, and PK parameters. We identified an effect of the route of administration on *V*_C_. The typical value for *V*_C_ was 0.141 L if administered IV and 0.043 L (ca. 70% smaller) if administered SC. Other significant covariate effects were not identified.

The between subject variability (BSV) for the PK parameters were described with exponential models. *K*_A_, clearance (CL), and peripheral volume of distribution (*V*_P_) have an estimated BSV of about 40% and the central volume of distribution (*V*_C_) of about 20% (Table 2). Because of the limited amount of data at low concentrations, the between subject variability and the individual predictions of the TMDD parameters were only estimated for the internalization rate *K*_INT_ (BSV: 49%). Model evaluation based on residual errors, OFV, standard errors, GOF plots, and individual curve fits corroborates that our final model adequately describes the PK of TAK‐079 in healthy monkeys (Table 2, Figure S4).

### 3.3 Pharmacodynamics

**Figure 3:**
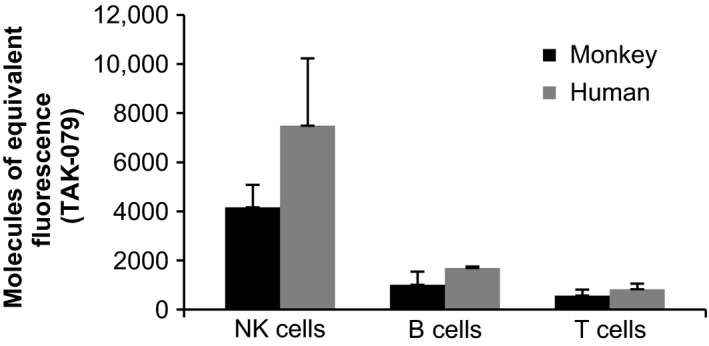
CD38 expression on the surface of human and monkey NK, B, and T cells. Direct comparison of CD38 expression levels on NK, B, and T cells in blood samples from monkeys (n = 3) and human subjects (n = 3). Monkey NK cells (CD3‐, CD159a+), B cells (CD3−, CD20+) and T cells (CD3+) and human NK cells (CD3−, CD16/CD56+), B cells (CD3−, CD19+) and T cells (CD3+) were measured using flow cytometry. The measurements are reported in MOEF

The level of TAK‐079 binding on human and monkey blood NK cells, T cells, and B cells was compared by flow cytometric analysis. As shown in Figure 3, monkey lymphocytes had CD38 expression levels, based on TAK‐079 MOEF, that were slightly lower compared to their human counterparts but with a similar relationship between cell types, for example, CD38 expression on NK cells > B Cells > T cells. These data support the use of this nonhuman primate species as a relevant model to help predict TAK‐079 potential for PD activity in humans.

For the in‐depth quantitative analyses of the relationships between drug exposure (PK) and the extent and duration of cell depletion (PD), we compiled data sets from PK concentrations, NK cell, B cell, and T cell counts of all 8 monkey studies, including the placebo‐treated animals, were available (Table 1). The initial characterization of the data set showed that at baseline, T cells had a median value of 3732 cells per μL (interquartile range (IQR): 2881‐5176) and were the most abundant lymphocyte subtype as compared to B cells with 1279 cells per μL (IQR: 860.8‐1890) and NK cells with 685 cells per μL (IQR: 482.8‐970.1). Baseline percentages of CD38+ cells in these cell populations was assessed in studies 1‐4 (Table 1, n = 67). 86.7% (SD 11.3) of NK cells expressed CD38 with little variability between animals. In contrast, 58.7% (SD 27.0) of B cells and 34.5% (SD 24.5) of T cells expressed CD38 with larger variability in the percentage of CD38+ cells in these lymphocyte populations.

Data from the placebo‐treated animals showed that the average number of each of the cell types varied over time between individual animals more than one would expect from the variability within one individual (Figure S5). For example, the average coefficient of variation in B cell counts of the individual placebo curves was 27% but the individual average B cell levels ranged from 436.6 to 4389 cells per μL. In addition, there were also differences between average baseline lymphocyte numbers from male and female animals and from animals of different studies adding to the variability (Figure S6). Based on these results, we chose to calculate for each posttreatment cell count the relative to its individual baseline value in percent, rather than the absolute cell numbers at each time point. For example, a value of 33% means that in this sample was 1/3 of the baseline cell count. This provided standardized values that could be compared across the entire data set.

**Figure 4:**
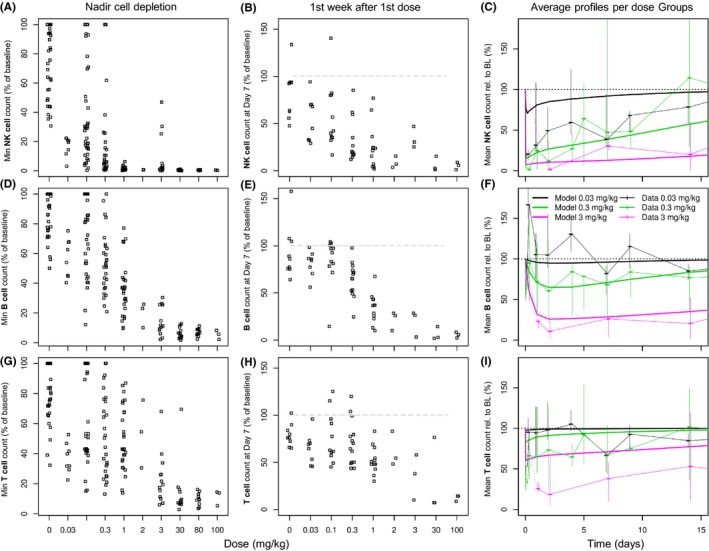
TAK‐079‐dependent NK cell, B‐cell, and T‐cell depletion. The graphs focus on changes after treatment with the first dose of TAK‐079. The data from single and multi‐dose studies with weekly or every other week dosing schedule were pooled. The upper row of graphs (A‐C) contains the individual minimal cell counts (i.e., the maximal PD effect), the individual cell counts 7 days after the first dose, and the average per dose cell depletion profiles overlaid by the model‐based population prediction of the NK cells (CD3‐/CD20−/CD16+) ‐ in this order. The middle row (graphs E‐F) contains the same information for the B cells (CD3−/CD20+) and the lower row for the T cells (CD3+) (graphs G‐I). For the graphs in the last column, the data of three IV dose groups were selected (after first dose, till day 14). Displayed are median and range of the data (% of baseline) at each sampling time point (thin lines) and the model‐based predictions (thick lines)

The rapid onset of depletion of TAK‐079‐binding cells suggests that the initial blood concentration drives the decrease in lymphocyte counts (Figure 4). At IV doses of 0.3 mg/kg TAK‐079, the median maximal effect on NK cells is depletion of 93.9% (i.e., 6.1% of baseline cell counts remaining). At 0.1 mg/kg, the peak depletion was 71%. At doses >0.3 mg/kg, NK cells were nearly completely depleted in the blood compartment (Nadir (range): 1.06 % of baseline (0.17, 6.23); Figure 4A). After a single dose of 0.3 mg/kg, it took approximately 7 days for the NK cells to recover to an average of 50% of baseline, albeit the kinetics of the recovery were highly variable between individuals (Figure 4B). In concordance with these results, NK function was also tested in a subset of animals in study 7 (n = 3/group; Table 1). This experiment showed a dose‐dependent reduction with minimal changes in blood NK activity at 48 hours posttreatment in animals treated with 0.1 mg/kg TAK‐079 (% lysis at 100:1 effector:target ratio ± SD; 44.5% ± 23.6% vs. 41.4% ± 25.8%) and almost complete loss of NK activity in animals treated with 1.0 mg/kg (% lysis at 100:1 effector:target ratio ± SD; 37.4% ± 10.3% vs. 6.8% ± 12.5%). NK cell function showed recovery at 57 days, the next time point measured (% lysis at 100:1 effector:target ratio ± SD; 16.0% ± 11.9%).

B cells and T cells were depleted to a lesser extent as compared to NK cells, which is consistent with their lower CD38 expression levels (Figure 3). For example, at 0.3 mg/kg IV TAK‐079, B cells had a median maximal level of depletion to 45% of baseline, and T cells were depleted to 43% of baseline (Figure 4D and G). At this dose level, a 50% reduction from baseline of B cell counts was not achieved in all animals. Only at the highest doses of ≥30 mg/kg were the B cells almost completely depleted (Figure 4D). T cells were depleted to an extent similar to B cells, but the recovery was faster (Figure 4E‐I).

In the two studies 7 and 8, we compared IV and SC dosing (Figure 4C,F and I). There were no obvious differences in cell depletion between the routes of administration. At the lower doses (study 8), a sustained (>24 hours) cell depletion of 50% below baseline values was only seen in the NK cell population and not with T and B cells; although all cells showed specific cell depletion at early time points. The timing of the onset of NK cell depletion appeared similar between dose groups regardless of the route of administration and the duration of depletion was dose‐dependent. Cell recovery in all test groups was seen by Day 57.

### 3.4 PK‐PD models

We developed separate PK‐PD models to describe the effects of TAK‐079 exposure on NK, B, and T cells. During PK‐PD modeling the PK parameters were kept fixed and a variety of PD models were evaluated (see Materials and Methods for detail). The NK cell population in the peripheral blood was adequately described with a turnover model and the depleting drug effect was linked via the PK concentration with an *E*_max_ type model to the rate of depletion. In this model the *E*_MAX_ represents the maximum rate of additional NK cell depletion (in addition to the base line rate of elimination *K*_OUT_) and the *C*_50_ the concentration at which the rate of additional NK cell depletion is half‐maximal. The structural PK‐PD model for NK cells was of the following form:

$$\frac{\mathrm{dNK}}{\mathrm{dt}}=K_{\mathrm{in}}-K_{\mathrm{out}}\cdot NK-NK\cdot \frac{E_{\mathrm{MAX}}\cdot c}{c_{50}+c}$$

In the formula, *NK* represents the actual NK cell count, *K*_IN_ the production rate and *K*_OUT_ the elimination rate when no drug is present. Note that with the given baseline measurement *BL, K*_OUT_ is defined by the equation *K*_OUT**_ *= K*_IN_ */BL*. *c* represents the TAK‐079 concentration in the central compartment. We achieved a successful estimation and reasonable goodness of fit with *K*_IN_ of 13 957 and an *E*_MAX_ of 415. The typical *C*_50_ estimate was 27.5 μg/mL (Table 3). Due to the limited differentiation between the maximal effects of the different doses (see previous section) and the large interindividual variability we could not expect accurate estimates of all parameters (Figure 4A‐C). For example, at the lowest dose of 0.03 mg/kg about 80% of NK cells are depleted at the first observation time point. This prevents an accurate description of the depletion dynamics at low doses at early time points. Consequently, the between subject variability was large with 111% for the NK production rate *K*_IN_ and with 146% for the *C*_50_, which is in accordance with the large individual differences at baseline and between‐treated animals. The model was evaluated based on residual errors, OFV, standard errors, GOF plots and individual curve fits (Table 3, Figure S7).

**Table 3:** PK‐PD modeling results: parameter estimates and standard errors in percent (%SE)

Parameter	Description	Final parameter estimate		Between subject variability/residual variability	
		Typical value (point est.)	%SE	Magnitude	%SE
NK Cells					
*K*_IN_ (count/day)	Production rate	13 957	4.52	111% CV	21.0
*C*_50_ (μg/mL)	Concentration of the half maximal effect	27.5	20.8	146% CV	24.5
*E*_MAX_	Maximal effect	414.6	10.4	NE	–
Baseline (NK cells)^a^	Baseline level	NE	–	28.6% CV	20.6
NK cells residual	Residual variability	–	–	0.2905	2.49
B Cells					
MTT (day)	Mean transit time	8.19	15.3	135% CV	17.6
*C*_50_ (μg/mL)	Concentration of the half maximal effect	19.8	8.95	NE	–
*E*_MAX_	Maximal effect	2.43	4.96	NE	–
Baseline (B cells)^a^	Baseline level	NE	–	24.03% CV	10.7
B cells residual	Residual variability	–	–	0.136	2.26
T Cells					
*C*_50_ (μg/mL)	Concentration of the half maximal effect	11.86	7.267	NE	–
*E*_MAX_	Maximal effect	0.4656	6.578	69.46% CV	29.50
Baseline (T cells)^a^	Baseline level	NE	–	29.08% CV	15.50
T cells residual	Residual variability	–	–	0.1343	2.406

CV, coefficient of variation; NE, Not Estimated.

a For each individual animal, the typical baseline value was calculated as average of all predose measurements.

The transit compartment model was superior to direct response or turnover models to describe TAK‐079‐induced B‐cell depletion. Four transit compartments turned out to be adequate and the drug effect was described with an Emax‐type model on the depletion rate. Like in the NK cell depletion model, the *E*_MAX_ represents the maximum rate and the *C*_50_ the concentration at which the rate is half‐maximal. Thus, the structural PK‐PD model for the B cells is given by the following five equations:

$$\frac{{\mathrm{dTR}}_{1}}{\mathrm{dt}}=K_{\mathrm{PROL}}\cdot {\text{TR}}_{1}-K_{\mathrm{TR}}\cdot {\text{TR}}_{1}$$

$$\frac{{\mathrm{dTR}}_{i}}{\mathrm{dt}}=K_{\mathrm{TR}}\cdot TR_{i-1}-K_{\mathrm{TR}}\cdot {\text{TR}}_{i},\text{for}\;i=2,3,4$$

$$\frac{\mathrm{dB}}{\mathrm{dt}}=K_{\mathrm{TR}}{\text{TR}}_{4}-K_{\mathrm{CIRC}}\cdot B-B\cdot \frac{E_{\mathrm{MAX}}\cdot c}{c_{50}+c}$$

*TR*_*i*_ (i = 1‐4) represent the four transit compartments. *K*_TR_ *, K*_PROL_ and *K*_CIRC_ are defined by the following equations *K*_TR_ *= K*_PROL_ *= K*_CIRC_ *= 4/*MTT, where MTT is the mean transit time.^9^ *B* represents the B‐cell count in the blood and *c* the TAK‐079 concentration in the central compartment.

The successful estimation resulted in the following point estimates (typical values) *E*_MAX_ 2.43, *C*_50_ 19.8 μg/mL and mean transit time (MTT) 8.19 days (Table 3). The delay of the maximal effect relative to the maximal TAK‐079 concentration was well captured (Figure 4F). The model indicates that TAK‐079 primarily affects circulating B cells. No additional effects and no feedback loop on progenitor cells were necessary to describe the available monkey B‐cell data. Between‐subject variability on MTT of 135% and on the baseline B‐cell levels (BASE) of 24% indicate large individual differences between animals.

The drug induced depletion of T cells with a rapid recovery was adequately described with a direct response model: *T(c)*_**_ = BL * (1−*E*_MAX_ * *c*/(*c *+ *C*_50_)), where *T* represents the actual T‐cell count, *BL* the T‐cell count at baseline and *c* the TAK‐079 concentration in the central compartment (Figure 4I). The typical *C*_50_ was estimated to be 11.86 μg/mL and the typical *E*_MAX_ was 0.47, indicating that in this case only about half of the T cells can be depleted by TAK‐079 (Table 3). Note however, that the between subject variability on *E*_MAX_ was nearly 70%. In this model, different from the NK and B‐cell depletion models, the *C*_50_ represents the concentration at which the depletion of T cells was half‐maximal. A special situation was observed in the 3 mg/kg group. Although the data at later time points are fitted adequately, the depletion after the first dose was underestimated (Figures 4I, S8). This is in accordance with observations from the repeated dose studies that, despite continuous treatment, T cells recover after initial depletion. In summary, the T‐cell model describes the data of the lower (clinically relevant) doses and of the repeated higher doses well but not the initial strong depletion after a first high dose.

Like for the NK cells, model evaluation of the final PK‐PD models for B and T cells based on residual errors, OFV, standard errors, GOF plots and individual curve fits corroborates that they adequately described the available monkey data (Table 3, Figure S7).

### 3.5 Simulation of human PK and cell depletion

The monkey PK and PK‐PD models were used as the starting point for the model‐based simulation of human PK and cell count data to support the design and to justify the selected doses for the FIH clinical trial in healthy volunteers. To this end, we assumed that the model structures including the accelerated clearance at low concentrations of TAK‐079 by TMDD derived from the monkey data also describe the primary features of the human PK and the ensuing lymphocyte depletion. To obtain predictions for the human parameters, we scaled the estimates of the following monkey PK parameters: central and peripheral volume of distribution (*V*_C_ *, V*_P_), clearance (*CL*) and intercompartmental clearance (*Q*) with a straight‐forward approach for monoclonal antibodies based on typical body weight values of monkeys and human subjects,^12^ Supporting Information. TAK‐079 is a fully human monoclonal antibody and, therefore, we expect less immunogenicity in humans than what was observed in monkeys. Consequently, for modeling and simulation we excluded ADA‐positive samples from the data set.

**Figure 5:**
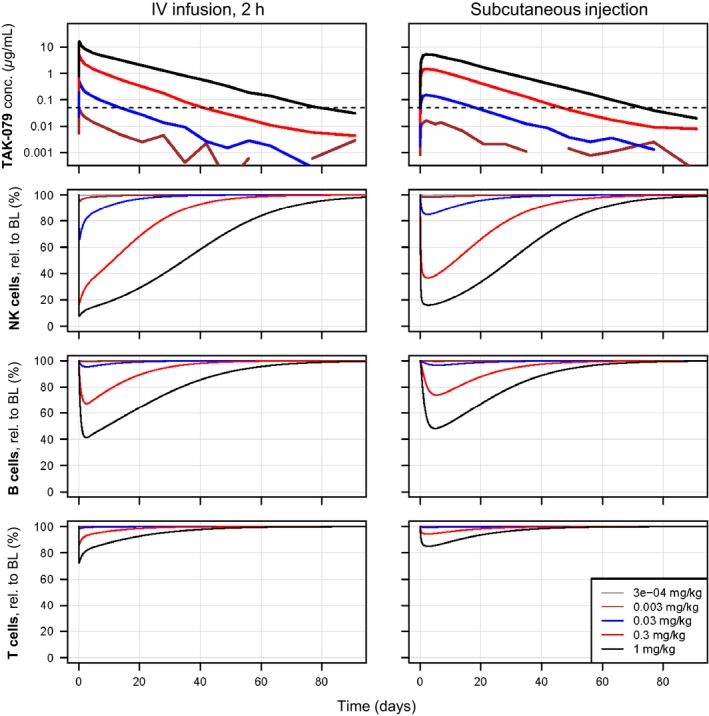
Simulated human PK and NK cell, B‐cell and T‐cell depletion profiles of TAK‐079. Based on the scaled monkey PK and PK‐PD models 5 single IV and SC dose PK and cell depletion profiles were simulated (from 0.0003 to 1 mg/kg). The left plots show the data after IV and the right plots after SC administration. The 2 plots in the first row display the PK profiles. The y‐axis is log scaled and the LLOQ of 0.05 μg/mL is indicated by a horizontal dashed line. The PK of the lowest dose was completely superimposed by noise and only at doses of 0.03 mg/kg the PK reaches levels above LLOQ

Using the scaled model, we simulated exposure and NK, B, and T‐cell depletion profiles for single doses [via a 2‐hour infusion (IV) or via SC injection] from 0.0003 to 1.0 mg/kg as planned for the FIH study (Figure 5). According to the simulations, after an IV dose of 0.0003 mg/kg, we would not expect any observable drug‐induced effects on lymphocyte counts and not even measurable PK concentrations above LLOQ. Due to the variability and limited size of the dose groups, we assumed that the minimal detectable drug effect on NK cell counts would be a reduction in at least 10%. At doses of 0.01 mg/kg IV and 0.03 mg/kg SC, we predicted that NK cells would be depleted to <90% of baseline.

At an IV dose of 0.3 mg/kg, we predicted NK cell depletion to 17% of baseline within 3 hours. After the end of infusion and recovery cells increased to more than 50% after 11 days (Figure 5). At the same dose, the model predicts that B cells are maximally depleted to 67% of baseline after 2.5 days and T cells are immediately depleted to 86% of baseline. For the subcutaneous administration of the same dose of 0.3 mg/kg, we predicted less and later maximal depletion (nadirs relative to baseline: NK cells 37%, B cells 74%, T cells 94%).

## 4 DISCUSSION

Our in vitro and in vivo preclinical studies demonstrate that the monkey is an appropriate animal model to study the pharmacology of TAK‐079. Densely sampled PK and cell count data of NK, B, and T lymphocytes from eight monkey studies with diverse doses and dosing regimen provide a rich data source for a comprehensive and quantitative understanding of the relationships between TAK‐079 dose, exposure, and cell depletion. The generated population PK and PK‐PD models adequately describe the observed data and provide a powerful tool to predict exposure and lymphocyte depletion not only for future studies in monkey but also for clinical trials in human subjects.

Our most important objective was to support the planning of the FIH‐single rising dose trial in healthy volunteers (http://www.clinicaltrials.gov: NCT02219256) without inducing a profound and lasting depletion of lymphocytes which could predispose them to infection. Therefore, we chose a conservative approach to determine the safe starting dose of 0.0003 mg/kg for the FIH trial.^6^, ^24^ Using data from preclinical studies,^22^, ^23^ we determined NK cell depletion as one of the most sensitive biological effects of TAK‐079. The PK‐NK simulation results helped us to identify the minimal dose level of 0.01 mg/kg IV at which we would expect the most sensitive pharmacological effect (NK cell depletion) to be detectable in humans.

Despite our rich database from 8 monkey studies, we recognized several limitations. TAK‐079 effectively depletes NK cells even at the lowest studied dose of 0.03 mg/kg. At such low doses, the PK quickly drops below the quantification limit of the bioanalytical assay, which prevents us from resolving the exposure‐effect relationship at lower doses. Moreover, during preclinical development, we recognized that maximal cell depletion occurs shortly after the maximal drug concentration but the resolution of the early phase of depletion is technically limited by the overall sample number and potentially by nonspecific cell depletion. Consequently, the power to accurately estimate the model parameters especially for NK cell depletion was limited.

We did not measure the effect of TAK‐079 on tissue plasma cells or plasmablasts, however, NK cells have high levels of CD38 on their surface and we could demonstrate that cell depletion efficiency of a specific lymphocyte subset depends, at least in part, on the expression levels of CD38. Therefore, we speculate that the cytolytic effect of TAK‐079 on plasmablasts and plasma cells is comparable to the effect on NK cells.^8^, ^19^, ^25^

The immunogenicity of TAK‐079 was assessed by measuring ADA levels. In the 13‐weeks repeated dose studies it was evident that over time more and more animals developed ADA and the levels increased. TAK‐079 is a fully human monoclonal antibody, and therefore we expect lower ADA levels in human subjects compared to what we observed in monkeys. Consequently, the information about the developing immunogenicity and its effects on drug elimination and potentially efficacy that can be gained in this animal model is limited.

With the emerging human data, it will be interesting to compare human and monkey PK and PD data in detail. The construction of a PK model based on human data and a comparison to the monkey model will allow refining the TMDD model of TAK‐079. Data generated in patient studies will provide insights how TAK‐079 mediated depletion of B lineage cells compares between healthy subjects and diverse groups of patients. The investigation of subject or disease‐related factors that may influence cell depletion efficiency in addition to CD38 expression levels is also important and could lead to a personalization of the treatment. In addition, a thorough head‐to‐head comparison of TAK‐079 with daratumumab and/or the other CD38 antibodies in vitro and in vivo will reveal valuable information about the pharmacology of anti‐CD38 antibodies and their optimal application.

The rich pharmacological data and the PK and PK‐PD models enabled us to characterize exposure‐effect relationships in monkeys. The model‐based analyses of NK, B, and T cells support and quantify the finding that each of the blood lymphocyte subsets are depleted by the antibody at different rates and require different time spans to replete the blood compartment. The models proved to be excellent means for simulations of PK and PD data under different dosing scenarios in preparation of monkey studies and the FIH clinical trial.

## AUTHORSHIP CONTRIBUTION

Participated in the design of monkey studies: GS, JY, LZ, SR; Modeling and simulation: GL, NP, SR; Performed data analysis: GS, JY, LZ, SR; Wrote or contributed to the writing of the manuscript: GL, GS, JY, LZ, NP, SR, ERF.

## DISCLOSURE

None declared.
