# Supplementary material for: Pharmacokinetics and pharmacodynamics of the cytolytic anti‐CD38 human monoclonal antibody TAK‐079 in monkey – model assisted preparation for the first in human trial
Source: Pharmacol Res Perspect. 2018 May 17;6(3):e00402. doi: 10.1002/prp2.402 (PMC5980133; doi:10.1002/prp2.402)
Supplement: Supplementary file 1 [file PRP2-6-e00402-s001.docx]

**Pharmacological characterization of the cytolytic anti-CD38 human monoclonal antibody TAK-079 in monkey – model assisted preparation for clinical trials**

Stefan Roepcke, Nele Plock, Josh Yuan, Eric R. Fedyk, Gezim Lahu, Lin Zhao, Glennda Smithson

Supplemental Information

Contents

[Supplemental Materials and Methods 2](#_Toc499645242)

[Data set preparation 2](#_Toc499645243)

[Scaling of monkey PK parameters 3](#_Toc499645244)

[Supplemental Tables and Figures 4](#_Toc499645245)

[PK data 4](#_Toc499645246)

[Population PK model evaluation 4](#_Toc499645247)

[PD data 9](#_Toc499645248)

[PK-PD models and model validation 10](#_Toc499645249)

[Berkeley Madonna simulation model code example for NK cells (exemplarily) 13](#_Toc499645250)

[References 14](#_Toc499645251)

## Supplemental Materials and Methods

**Table S1.** Antibodies used for flow cytometric analyses in PD studies

| **Antibody** | **Antibody volume per sample (µl / 100 µl sample)** | **Vendor** |
| --- | --- | --- |
| CD3-APCH7 (SK7)* | 5 | BD Biosciences |
| CD3-APC Cy7 (SP34-2) | 1.25 | BD Biosciences |
| CD3-PerCp Cy 5.5 (SP34-2) | 5 | BD Biosciences |
| TSF-19- AF647 | 1 | Prepared at Takeda |
| TAK-079-AF488 | 2 | Prepared at Takeda |
| TSF-19- AF488 | 0.42 | Prepared at Takeda |
| CD19-PerCp Cy 5.5 (HIB19)* | 5 | BD Biosciences |
| CD20-PE (2H7) | 10 | BD Biosciences |
| CD20 –APCH7 (2H7) | 2.5 | BD Biosciences |
| CD16-PE (B73.1)* | 5 | BD Biosciences |
| CD16-PerCP-Cy5.5 (3G8) | 20 | BD Biosciences |
| CD56-PE (B159)* | 5 | BD Biosciences |
| CD159a-PE (Z199) | 5 | Beckman Coulter (Brea, CA) |
| CD45-PerCP TruCount™ (DO58-1283) | NA | BD Biosciences |
| CD45-PeCy7 (HI30)* | 2.5 | BD Biosciences |
| CD45-PeCy7 (DO58-1283) | 2.5 | BD Biosciences |
| Mouse IgG1 kappa -AF488 or -AF647(MOPC-21) | 2.5 | BioLegend (San Diego CA) |

***used for staining human cells only**

### Data set preparation

The data sets from the 8 monkey studies were collected, reorganized in a single format and merged in to three separate NONMEM readable PK-PD data sets. Each of the three data sets contained individual characteristics of the monkeys (study, ID, group, body weight, sex), the dosing information, the PK and either NK, B, or T cell data. For animals of the control groups only cell counts but no PK data were added to the data sets, assuming implicitly no serum levels of TAK-079. Time-resolved information about the anti-drug immunogenicity status (ADA), namely TITER containing the quantitative measurement result and the 0/1-flag variable ADAF (ADAF=1 if ADA affects the concentration of TAK-079, ADAF=0 if it does not), was added in separate columns to each observation. ADA titers were measured with different method specifications in the different studies and, therefore, between studies the values are quantitatively not directly comparable. To utilize the ADA information in a consistent manner across all studies we applied the following procedure for each animal separately: ADA titers that increased at time points later than 7 days over the initially measured levels were considered ADA-positive and flagged in the data set (ADAF=1). If a sample at one time point was flagged ADA-positive all samples that were taken after that time point were also flagged ADA-positive in this animal regardless of the measured titer. ADA positive observations were not used for parameter estimation during model development. Note that also PD measurements from sampling time points of ADA affected PK concentrations were flagged with ADAF=1.

For the cell count data, the individual baseline values for each cell type (NK, B, and T cells) were calculated as mean value of all available predose measurements of a given animal. In most studies, this was a single measurement. The baseline value of each animal was then added as an observation at the time of the first dosing event (TIME=0) and as a constant value in column BL to each observation of the respective animal. Based on this baseline value the percent of baseline for each observed cell count was calculated and added to the data set.

### Scaling of monkey PK parameters

The final PK and PK-PD models were used as starting point to simulate PK and PK-PD profiles for the first in human clinical trial. Comparative analyses of data from therapeutic monoclonal antibodies have shown that PK parameters derived from studies in monkeys can be scaled to predict human PK profiles with acceptable accuracy (Han & Zhou, Therapeutic Delivery, 2011). The publication indicated that using a fixed exponent of 0.85, human clearances of monoclonal antibodies can be predicted reliably. Consequently, this relationship was applied to scale human clearance parameters (CL, Q), whereas volume parameters (V_C_, V_P_) were scaled using a direct relation between body weights (BW), assuming typical body weights of 2.6 kg for monkeys and 70 kg human subjects:

$$\begin{matrix} CL_{human}=CL_{animal}\cdot\left(\frac{BW_{human}}{BW_{animal}}\right)^{0.85} \\ V_{human}=V_{animal}\cdot\frac{BW_{human}}{BW_{animal}} \end{matrix}$$

## Supplemental Tables and Figures

### PK data

Based on the data of all monkey studies excluding the two lowest dose groups (dose >0.3 mg/kg) a linear 2-compartment model was constructed. When we simulated the PK of the lowest dose groups and overlaid the measured concentrations it was evident that the linear model over predicted the concentrations (Figure S2).

**Figure S2.** Linear PK models overpredict low PK concentrations

**
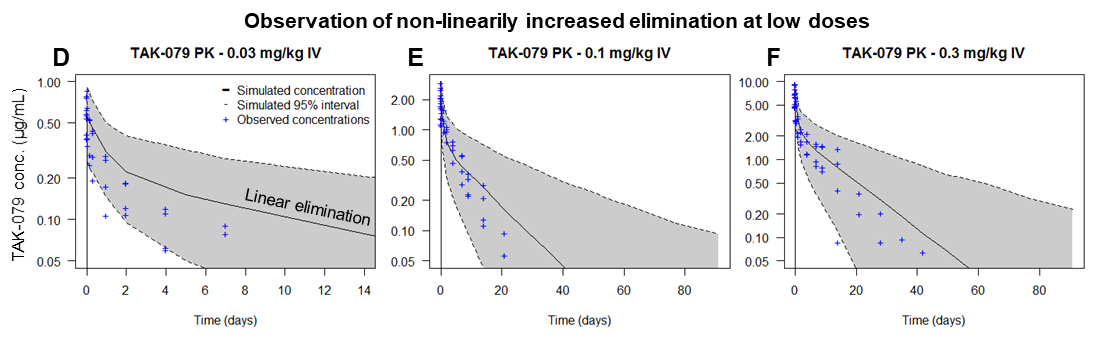
**

**ADA** was detected with a validated ECL assay. The incidence increased over time and affected PK when it reached a specific threshold titer of circa 1000 (~log(7)).

**Figure S3.** ADA effects in 13-week toxicology study


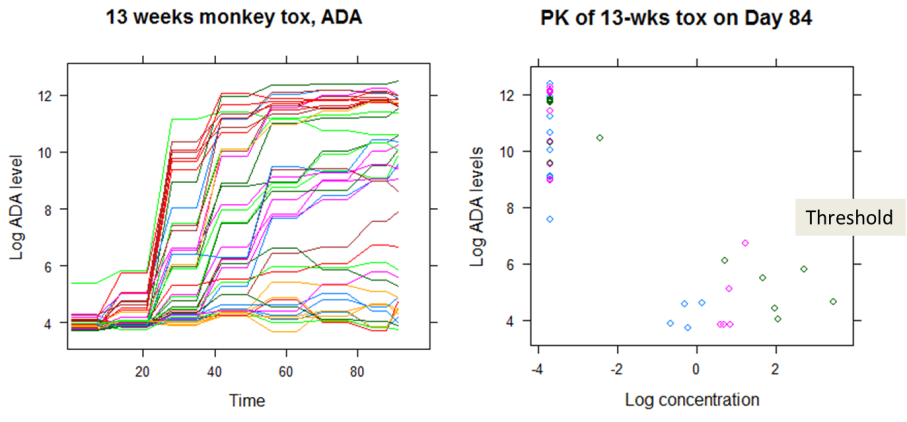


### Population PK model evaluation

The evaluation refers to the final population PK model (Figure 2, Table 2). Presented are the following goodness-of-fit (GOF) plots stratified by dose and route of administration (Keizer et al., 2013):

1. Conditional weighted residuals (CWRES) versus time
2. Observed concentration versus population model prediction
3. CWRES versus population model prediction
4. Observed concentration versus individual model prediction

**Figure S4.** GOF plots for the final population PK model stratified by dose and route of administration (IV - red, SC - blue)


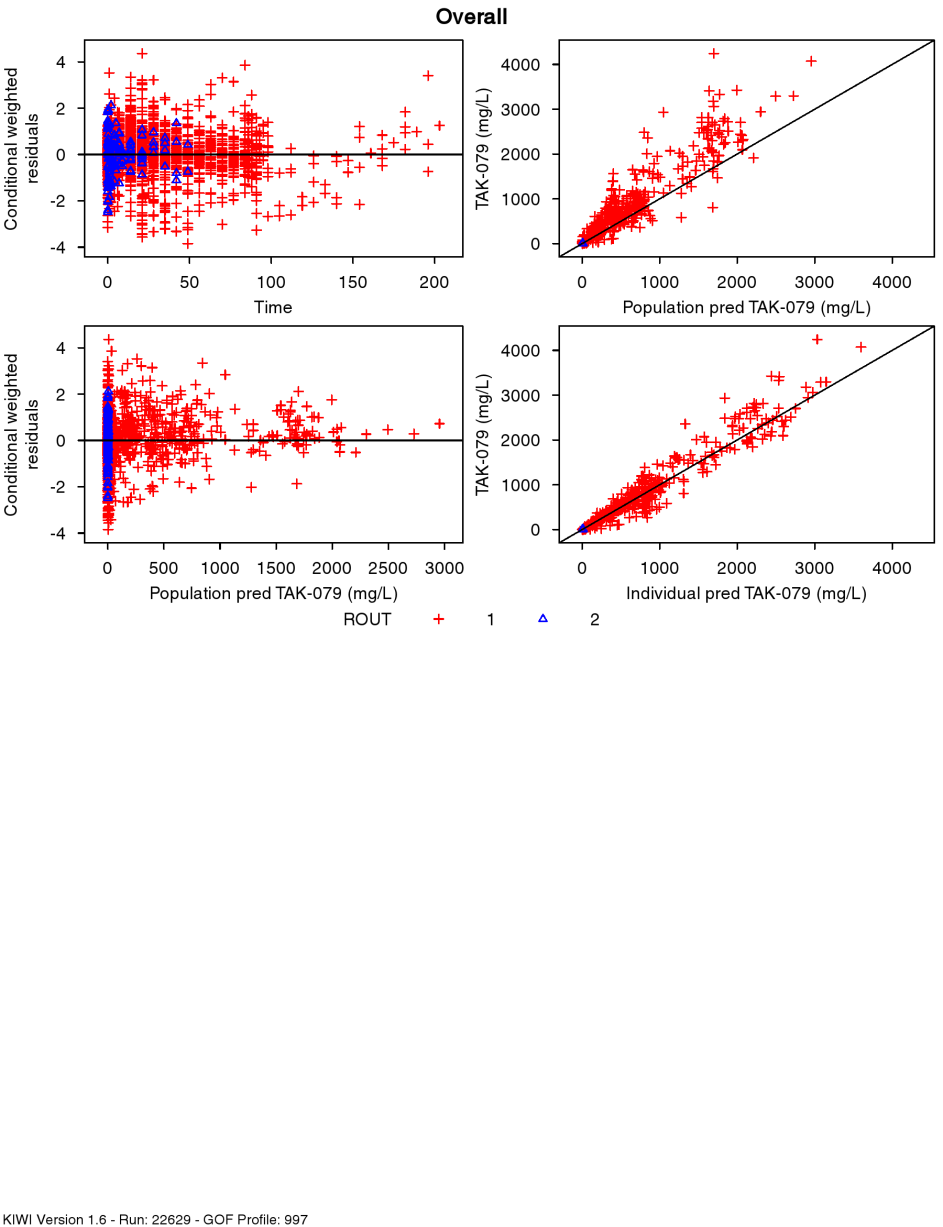

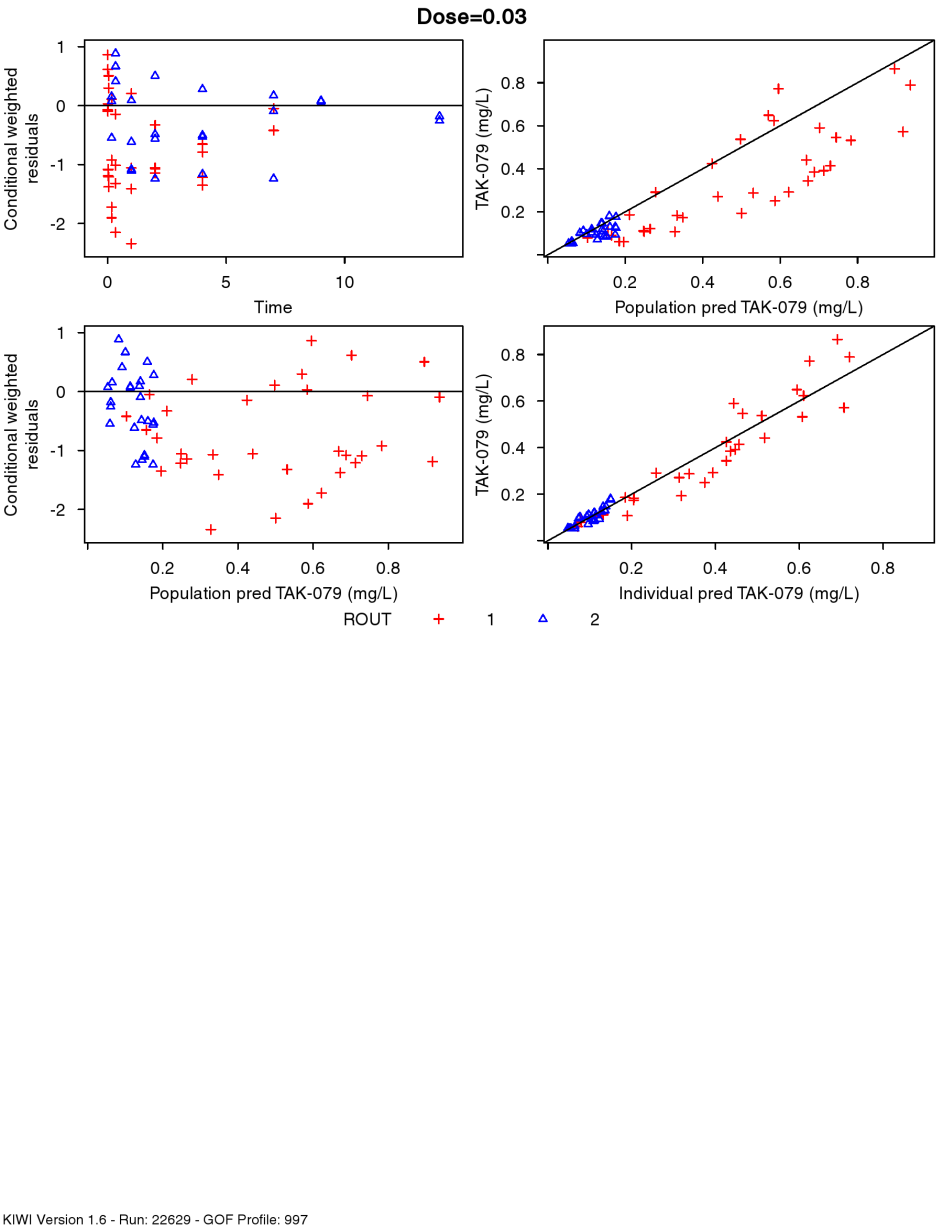

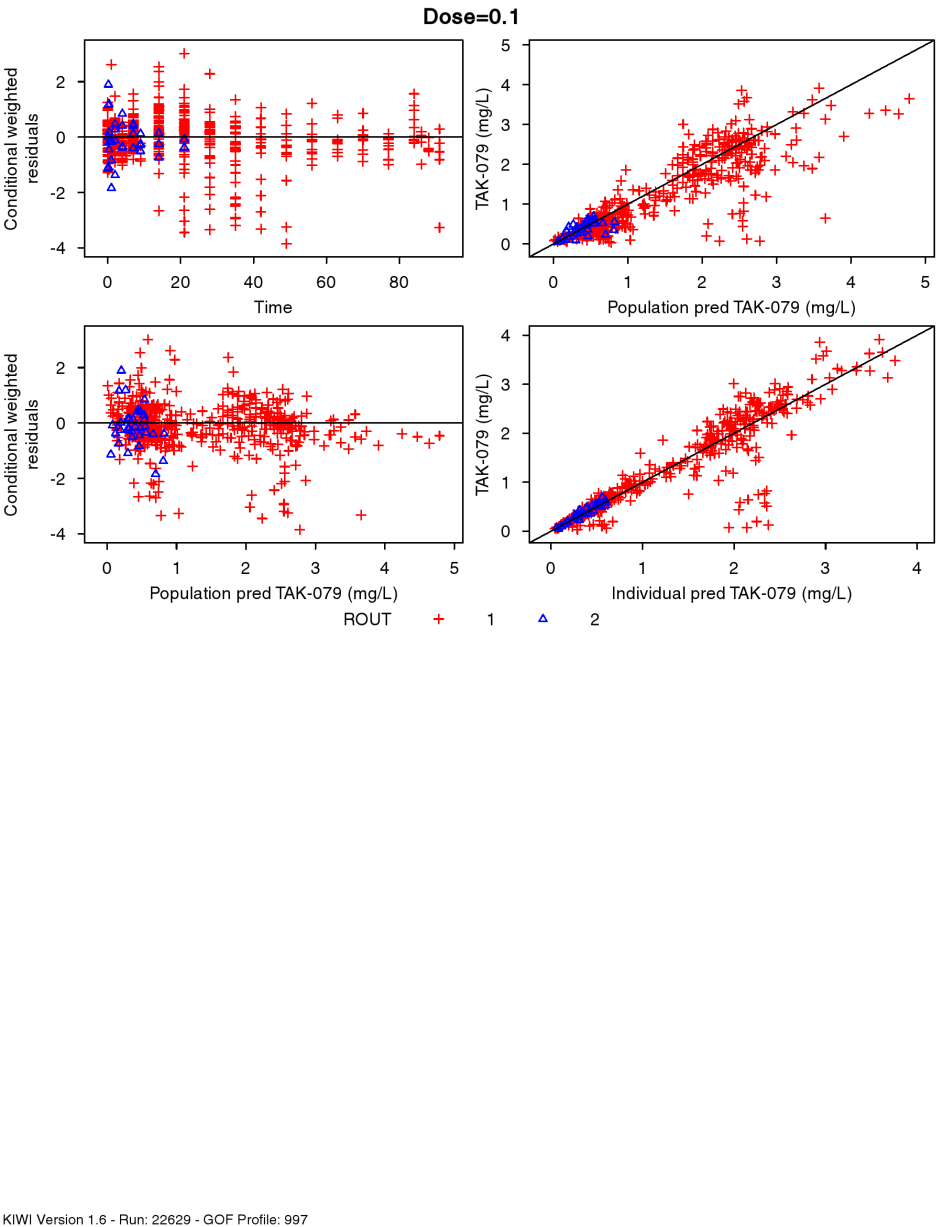


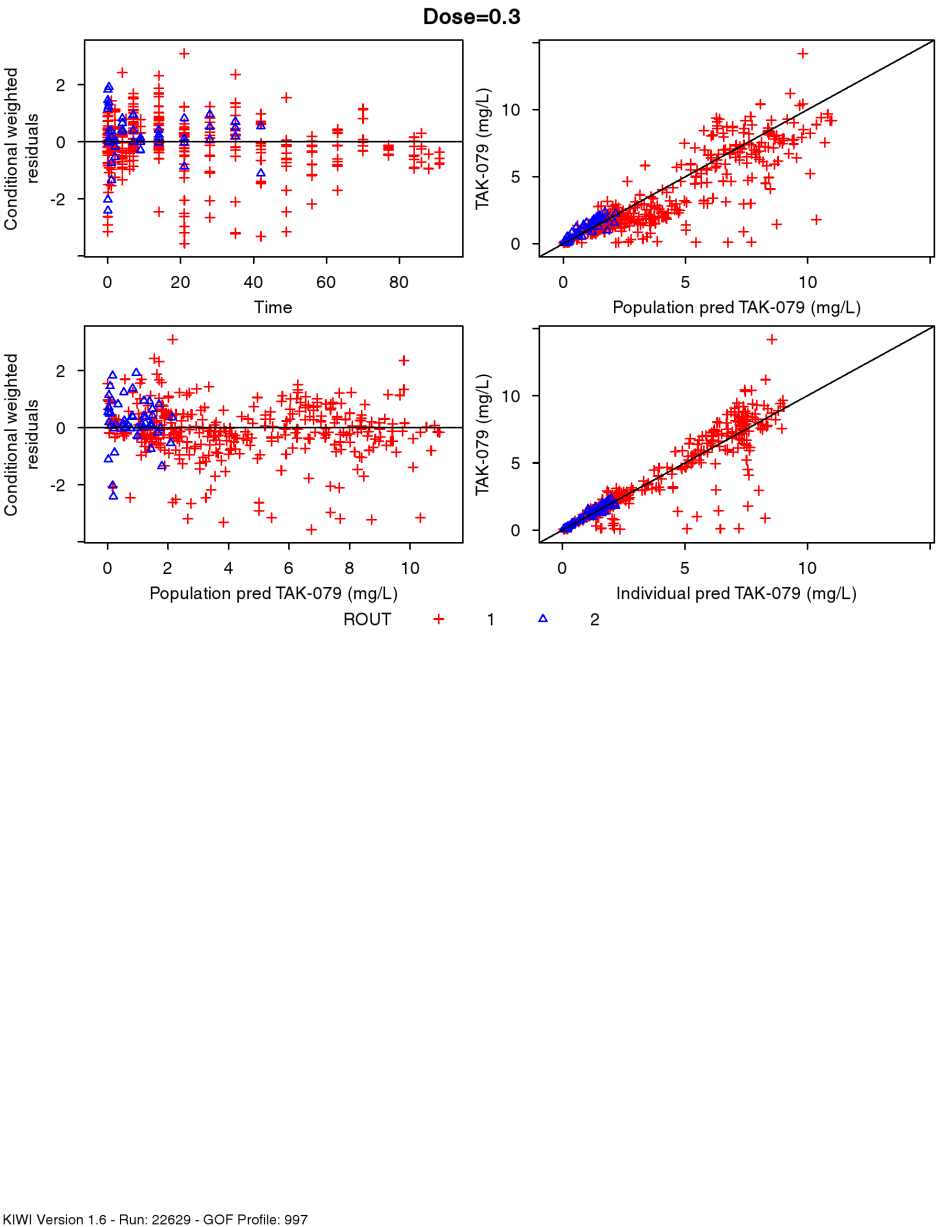

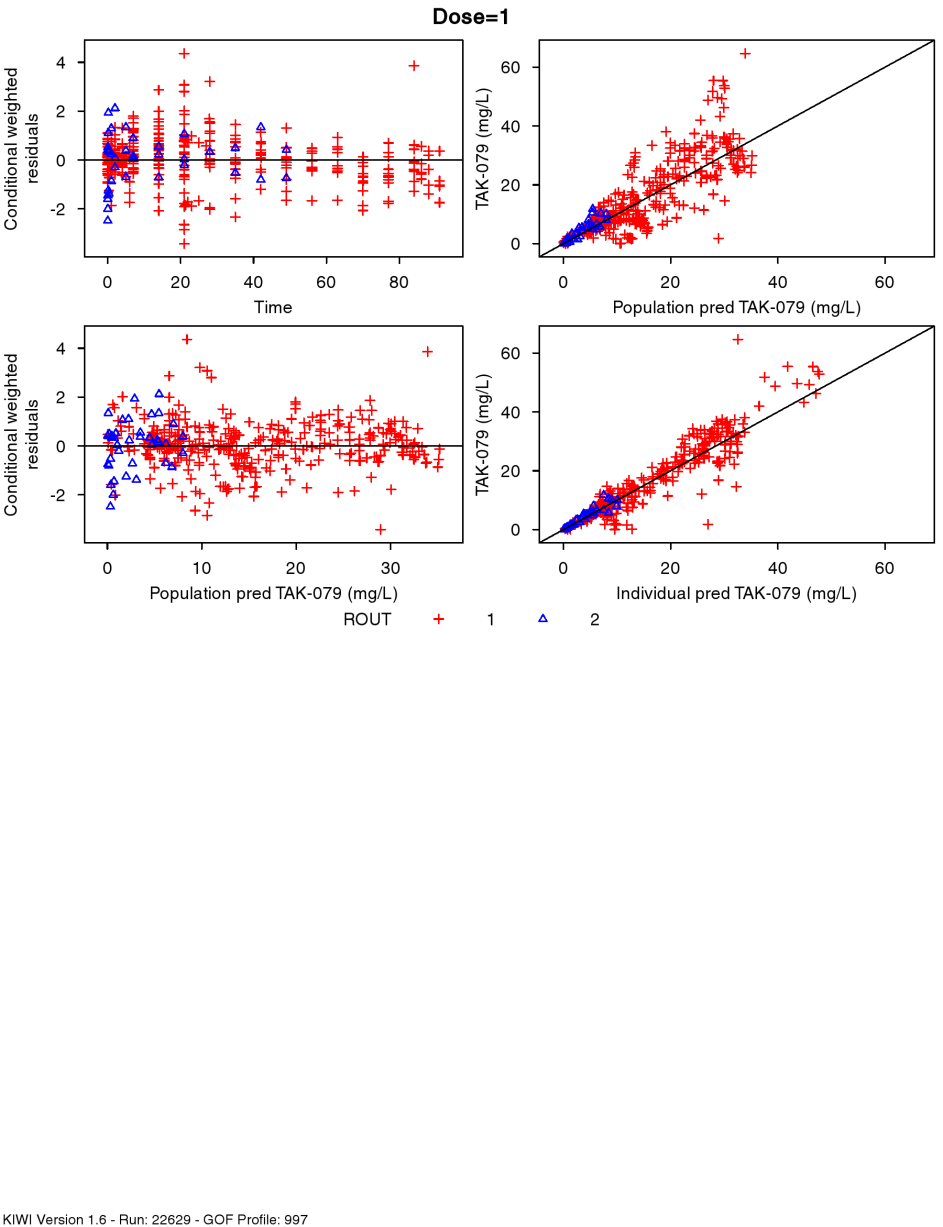

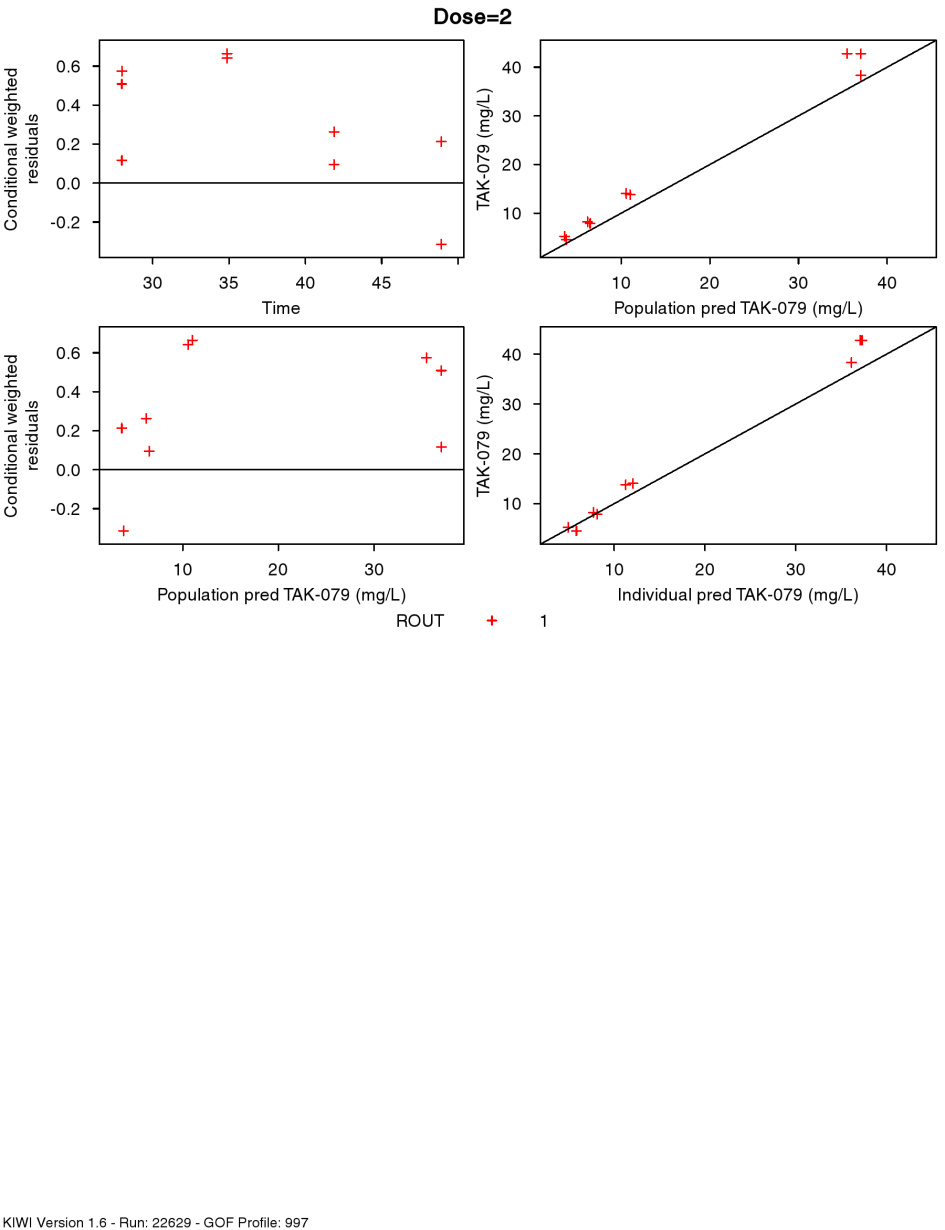

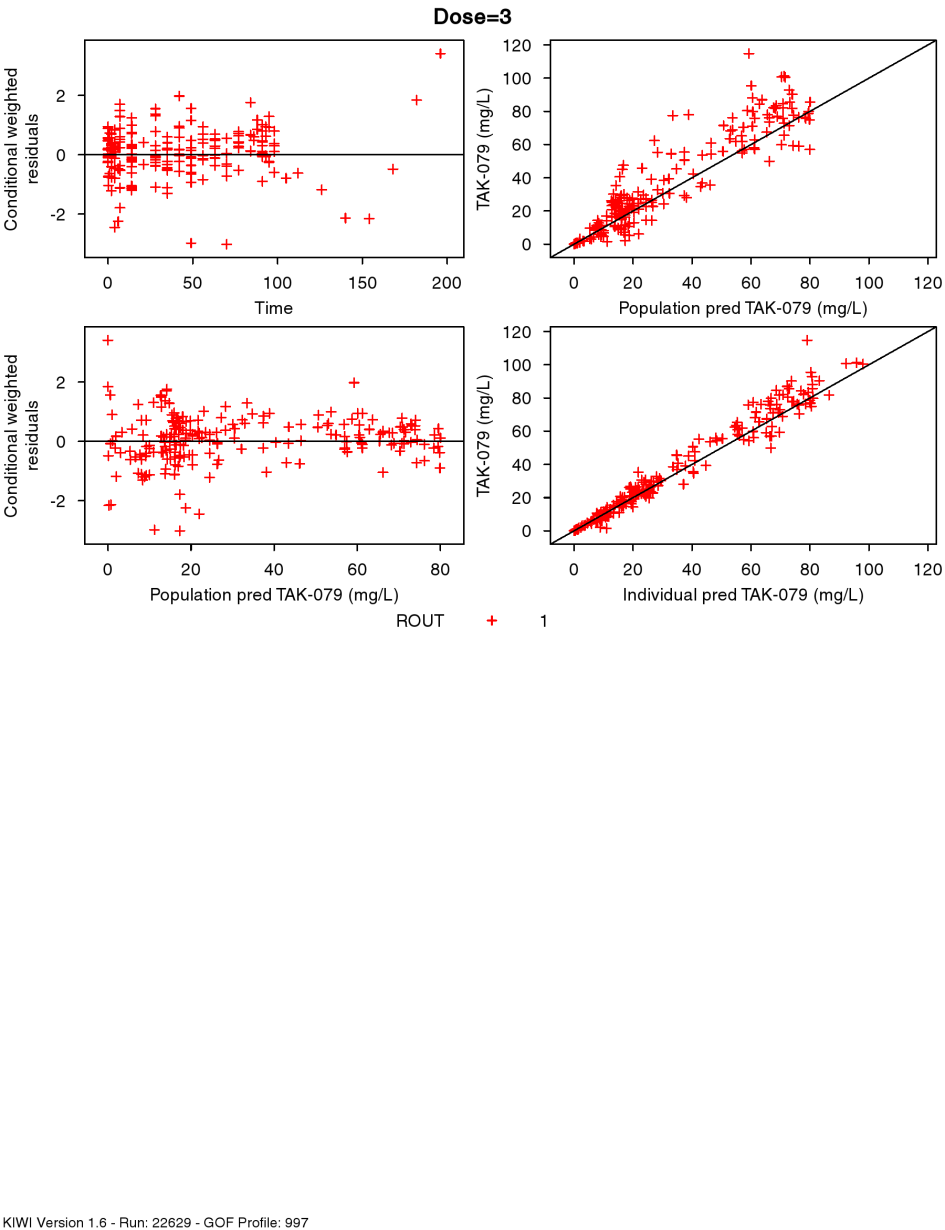

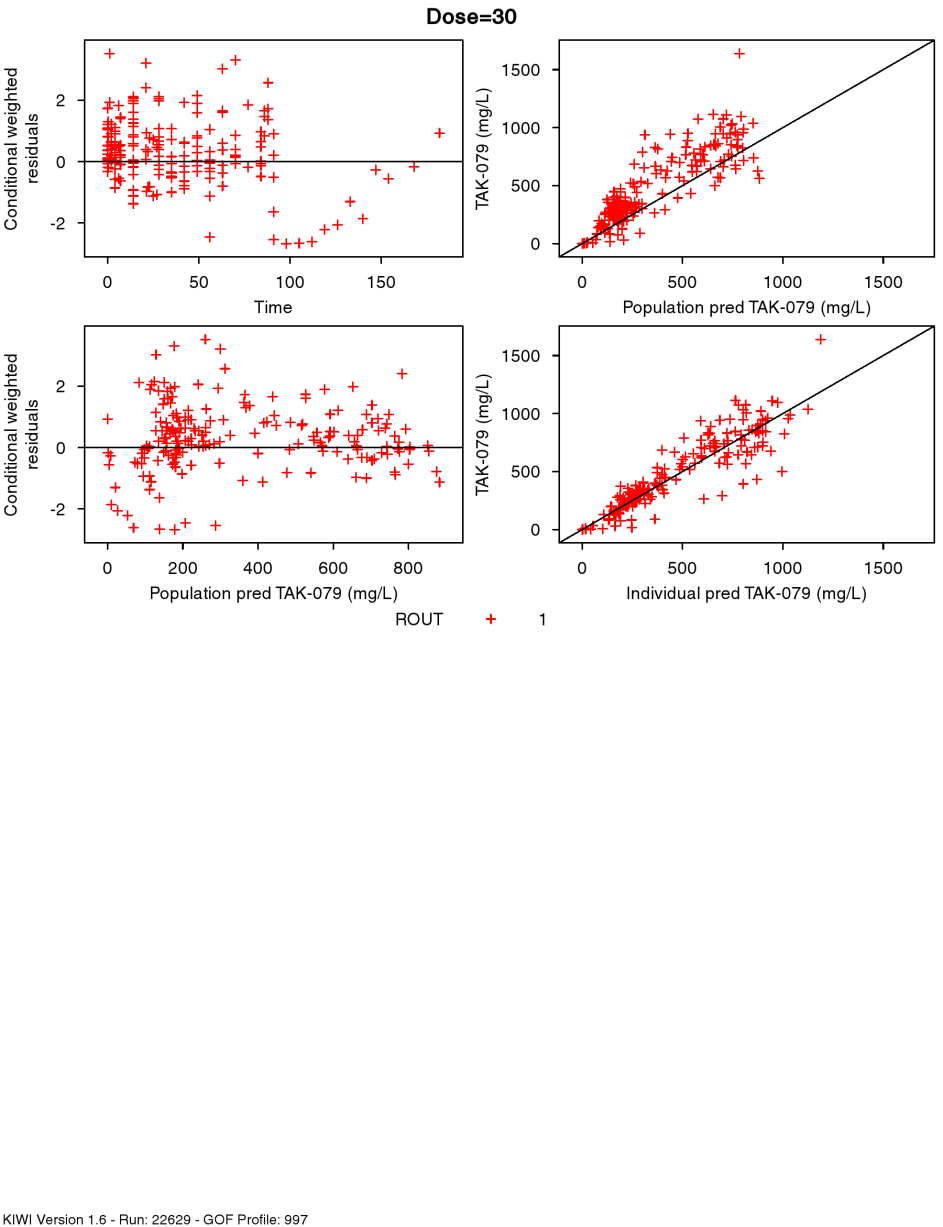

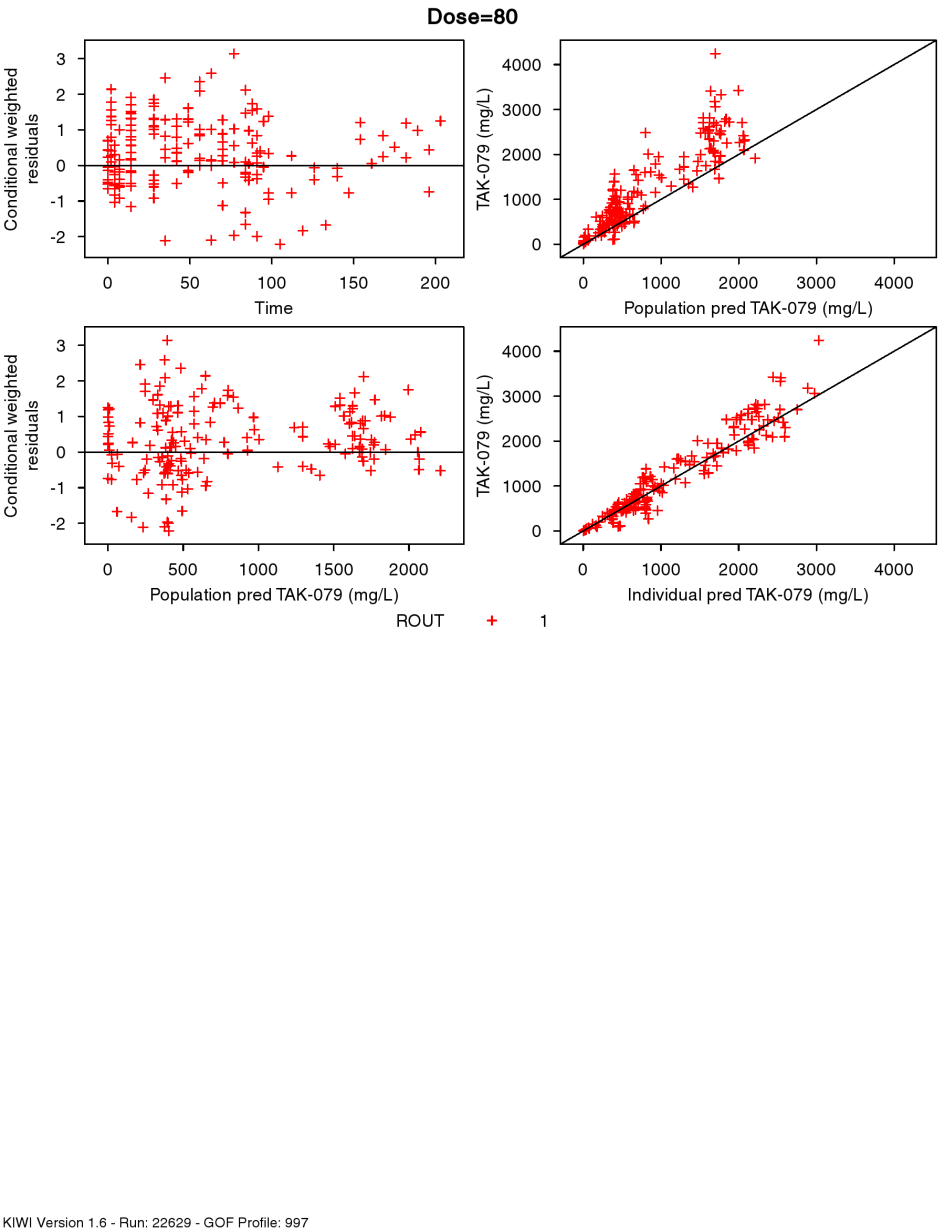

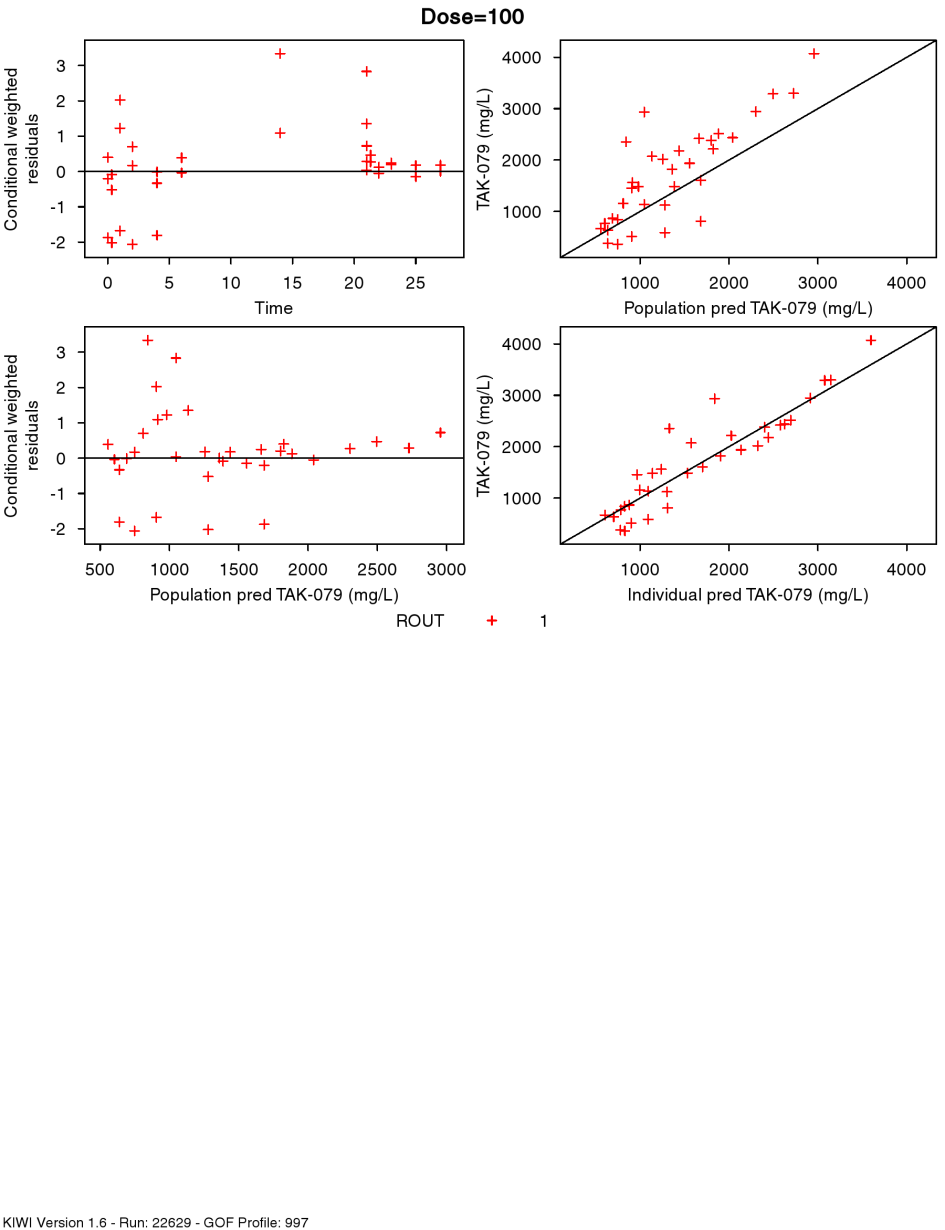


### PD data

**Figure S5.** Inter- and intra-individual variability in the T cell, B cell and NK cell count data of the placebo treated animals


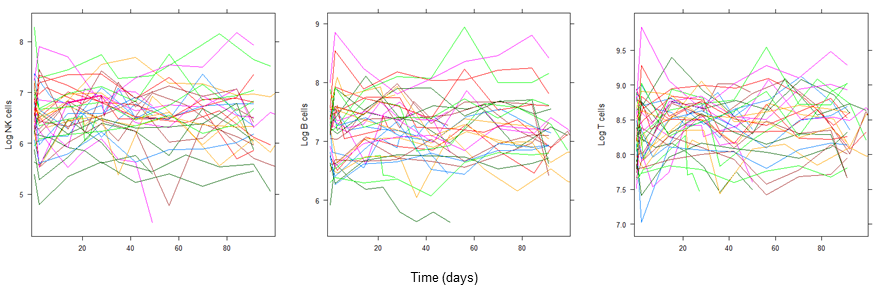


**Figure S6.** Predose NK, B, and T cell counts (cells per µL) stratified by study (upper row) or sex (lower row)


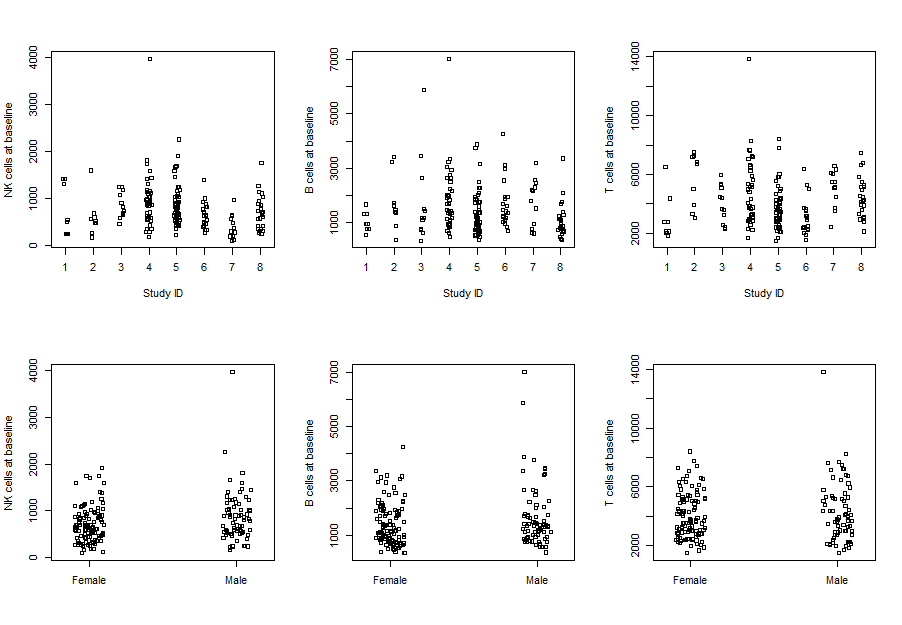


### PK-PD models and model validation

**Structures of PK-NK cell, PK-B cell, and PK-T cell models**

*K_IN_*

NK Cell
counts

*K_OUT_*

B Cell
counts

TR1

TR4

TR3

TR2

*K_CIRC_*

*K_PROL_*

*K_TR_*

*K_TR_*

*K_TR_*

*K_TR_*

The evaluation refers to the final population PK-PD model (Table 3). Presented are the following goodness-of-fit (GOF) plots stratified by route of administration (Keizer et al., 2013):

1. Conditional weighted residuals (CWRES) versus time
2. Observed concentration versus population model prediction
3. CWRES versus population model prediction
4. Observed concentration versus individual model prediction

**Figure S7.** GOF plots for PK-PD models, stratified on route of administration (IV – red, SC – blue)

NK cells


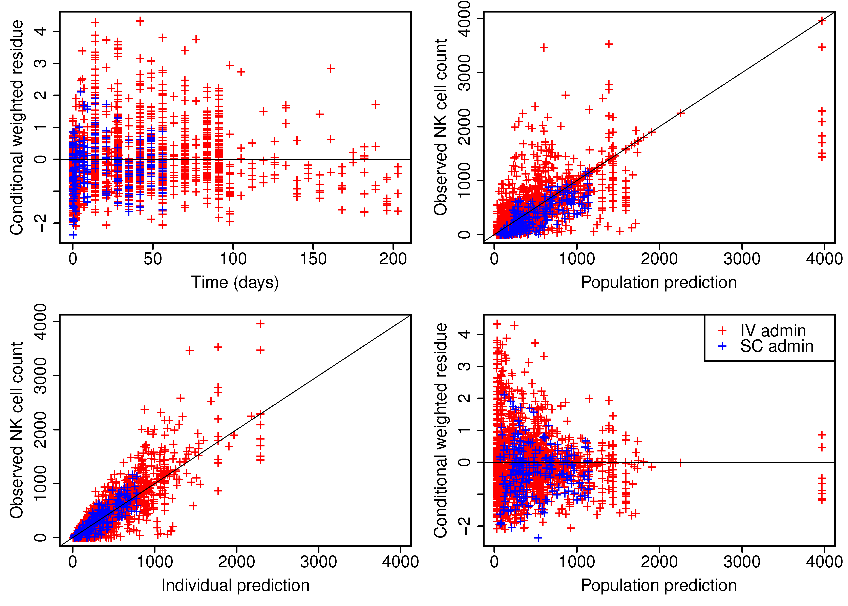


B cells


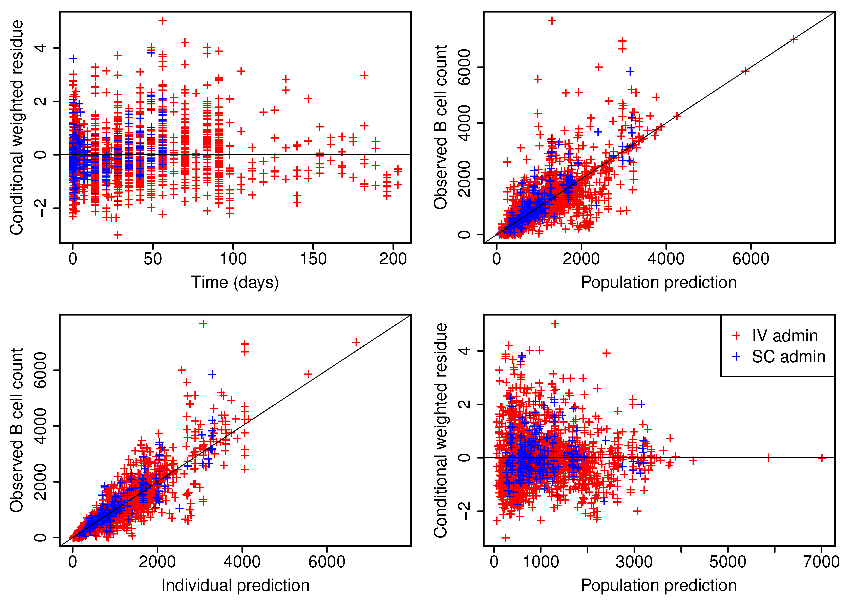


T cells


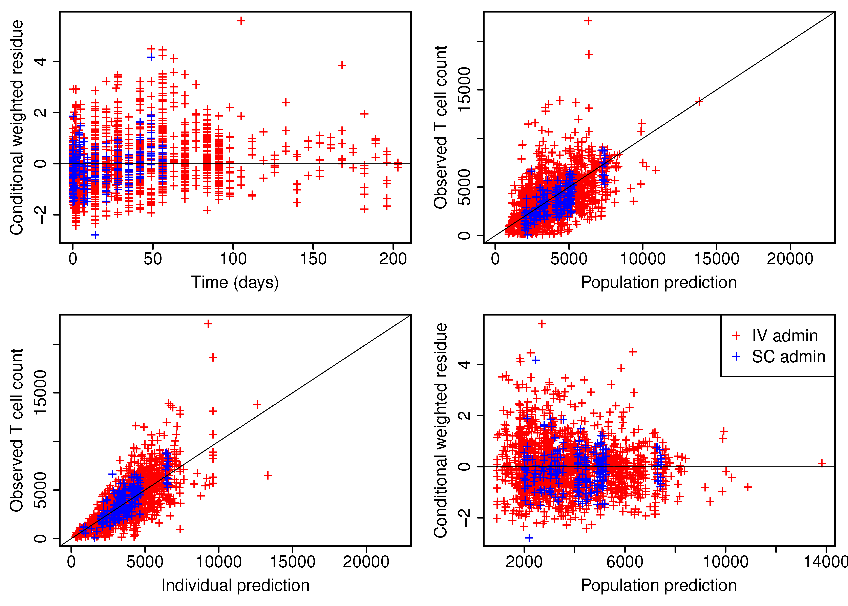


**T cell recover despite continuous treatment**

T cells are the most abundant lymphocyte subgroup. They express less CD38 on their surface and they are depleted to a lesser extent by TAK-079 compared to NK and B cells (Figures 3, 4 G-I). The direct response model fits the data of the low dose groups and at later time points adequately. In the high dose groups, however, the depletion after the first dose was underestimated (Figures 4 I, S9). For example, in the 3 mg/kg dose group, the individual data and the weighted residues (CWRES) suggest a strong response (cell depletion) after the first dose and subsequent recovery of the T cell population regardless of the continued dosing.

**Figure S8.** T cells data of single and repeated 3 mg/kg dose groups, individual profiles (left) and conditionally weighted residues (right)


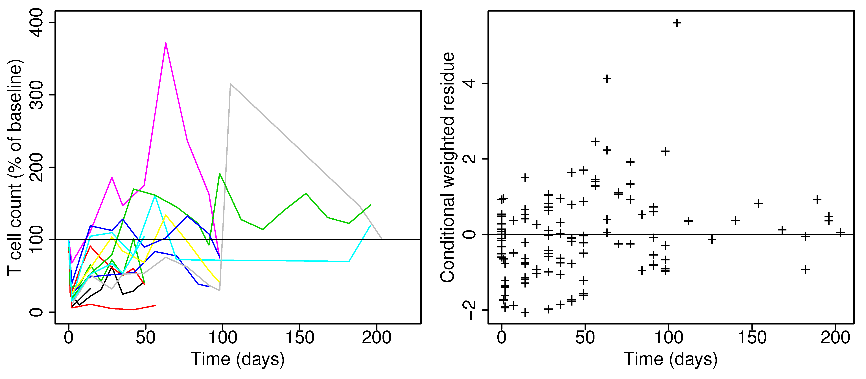


### Berkeley Madonna simulation model code example for NK cells (exemplarily)

METHOD RK4

STARTTIME = 0

STOPTIME =100

DT = 0.01

DTOUT=0.1

init depot = SQDOSEBW

init central = 0

init periph = 0

init rtot = RBASE

init nkcell = BL

init (AUC)=0

;--------PK MODEL--------

CONC=central/V1

K10=CL/V1

K12=Q/V1

K21=Q/V2

RBASE = KSYN/KDEG

d/dt (depot) = -KA * depot

d/dt (central) = - K12*central + K21*periph - K10*central - KINT*rtot*central / (KSS+CONC) + KA * depot

d/dt (periph) = - K21*periph + K12*central

d/dt (rtot) = KSYN-KDEG*rtot - (KINT-KDEG)*rtot*CONC/(KSS+CONC)

;-------- NK CELL PK-PD MODEL--------

KOUT=KIN / BL

EFF=EMAX * CONC / (EC50 + CONC)

d/dt (nkcell) = KIN - KOUT * nkcell - nkcell* EFF

nkcell_percent=(nkcell/BL)*100

d/dt(AUC)=central/V1

;-------- PARAMETERS --------

F1 = 0.5565979 ;;exp(x) / (1 + exp (x)), x=0.227366

KA = 0.398734

CL= 0.0187399 *(70/2.6)**0.85

V1= (0.140556 *(70/2.6)) * (1 - 0.696753)

Q = 0.127358 * (70/2.6)**0.85

V2= 0.127326 *(70/2.6)

KINT = 0.1

KSS = 5.68

KSYN = 0.04

KDEG = 0.00452

BL=685

KIN=13957

EC50=27.5

EMAX=414.6

;--------DOSE--------

BW=70

DOSE=0.3 ;mg/kg

SQDOSEBW = F1 * DOSE * BW ;mg

## References

Keizer RJ, Karlsson MO and Hooker A (2013) Modeling and Simulation Workbench for NONMEM: Tutorial on Pirana, PsN, and Xpose. *CPT Pharmacometrics Syst Pharmacol* **2**:e50.
